# Toxicity of phthalate esters to lettuce (*Lactuca sativa*) and the soil microbial community under different soil conditions

**DOI:** 10.1371/journal.pone.0208111

**Published:** 2018-12-20

**Authors:** Tingting Ma, Wei Zhou, Like Chen, Longhua Wu, Peter Christie, Wuxing Liu

**Affiliations:** 1 Key Laboratory of Original Agro-Environmental Pollution Prevention and Control, Ministry of Agriculture / Tianjin Key Laboratory of Agro-environment and Safe-product, Tianjin, China; 2 Institute of Hanjiang, Hubei University of Arts and Science, Xiangyang, China; 3 Key Laboratory of Soil Environment and Pollution Remediation, Institute of Soil Science, Chinese Academy of Sciences, Nanjing, China; 4 School of Civil Engineering and Architecture, Hubei University of Arts and Science, Xiangyang, China; 5 Shanghai Research Institute of Chemical Industry, Shanghai, China; Nanjing University, CHINA

## Abstract

Phthalate esters (PAEs) are globally used plasticizers and typical endocrine disruptors that can readily accumulate in agricultural products and represent a substantial risk to human health via the food chain. The range of soil properties has an important influence on the expression of PAE toxicity, and the mechanisms by which soil physical and chemical properties affect the expression of toxicity of target PAEs to plants and microorganisms requires further investigation. Important soil factors affecting the eco-toxicological effects of two typical PAEs, di-n-butyl phthalate (DnBP) and bis (2-ethylhexyl) phthalate (DEHP), on lettuce (*Lactuca sativa*) in a spiked soil were investigated in the present study. Soil at various pH values was spiked with three PAE concentrations (1, 5 and 20 mg DnBP or DEHP kg^-1^ soil), organic matter contents and water holding contents to simulate the greenhouse soil environment for 30 days. Their influence on the biomass, photosynthetic pigment contents, various physiological changes and soil microbial communities was determined as endpoints. The toxicity to lettuce of DnBP was higher than that of DEHP in the soil and soil pH was the most important factor affecting their single toxicity, followed by soil organic matter content and soil moisture content in agreement with the Biolog test results. Under different soil conditions total protein, total soluble sugar and free amino acid contents were positively correlated with concentrations of the target PAEs, but leaf area, biomass, •O_2_^-^ activity, vitamin C content and soil microbial diversity indices showed the opposite trend. Chlorophyll a and carotenoid contents were more inhibited by DnBP together with impacts on indices of soil microbial diversity. The results suggest that soil conditions in greenhouses directly explain the patterns of pollutant toxicity displayed and impact the quantity, quality and food safety of vegetables produced using highly intensive production systems.

## Introduction

Phthalate esters (PAEs) are a class of synthetic organic compounds with important environmental hormone-like activities and are the most commonly used plastic additives used to increase the flexibility of plastic polymers [1]. PAEs are present in thousands of industrial, medical, agricultural, and domestic products such as fabrics, surfactants, detergents, mulching membranes, toys, wallpaper, medical tubes, wrappers and personal care products [2,3]. They are combined physically rather than chemically with the polymer matrix so that PAE compounds can be readily released into the atmosphere, water, soil, sediments and foods in relatively large quantities because of their large-scale and worldwide use [2,4–6].

Di-n-butyl phthalate (DnBP) and di (2-ethyl hexyl) phthalate (DEHP) are the two most frequently detected PAE additives and both are included in the priority list produced by the US Environmental Protection Agency (USEPA) as potential endocrine disrupting compounds (EDCs) [7] inducing oxidative stress, genotoxicity, inflammatory and reproductive diseases, and cancers [8–12]. DnBP and DEHP are commonly detected in greenhouse and vegetable soils, especially where plastic films are used, and total concentrations of > 32 mg kg^-1^ have been recorded in Jiangsu, Guangdong and Shandong provinces and other urban areas in China [13–21]. Over 2.5 million tonnes of agricultural plastic films have been used annually up to 2015 in China. Soils are the main environmental compartment acting as major sinks for pollutants in intensively managed cropping systems and the control of soil PAE contamination has become a topic of great concern in China.

Uptake and accumulation by plants is an important pathway in the migration and transformation of PAEs in the environment. The uptake of EDCs by plants is of great concern due to their potential to accumulate and to their long-term effects [19,22,23]. In 11 vegetable species collected from nine farms in the Pearl River Delta, south China, the concentrations of six PAEs ranged from 0.073 to 11.2 mg kg^-1^ (dry weight/DW basis) [24]. At e-waste disassembly sites the concentrations of six PAEs in *Vicia faba* [14.01 ± 2.01 mg kg^-1^ DW] were significantly higher than in cabbage or rape [25]. In vegetables cultivated on land surrounding a polymer production plant the maximum DEHP contents in *pak choi*, field mustard, eggplant, and cowpea were 52.0 ± 3.1, 43.1 ± 2.2, 36.2 ± 2.8, and 19.4 ± 0.47 mg kg^-1^ DW, respectively, leading to concern over food safety on the basis of the daily intake of these vegetables cultivated within 200 m of the polymer production building [26]. PAE concentrations in soil and soybean with large amounts of mulch residues were markedly higher than with low residues, and a significant correlation between PAE content and plant growth period was derived [27,28].

Soil quality and productivity may be affected by contamination with PAEs. There is increasing concern because of the inhibition of soil microbial community functional diversity, microbial biomass carbon, soil basal respiration and the activities of various soil enzymes due to high concentrations of DnBP and DEHP in soils [29–32]. Abnormal cell division, growth delay, inhibition of photosynthesis, and yield reduction of vegetables are often caused by PAEs [33–37] together with an increase in micronucleus rate, oxidative damage and genotoxicity in *Vicia faba* [38]. Inhibition of germination, abnormal root morphology, increasing membrane permeability and antioxidant levels, decreasing soluble sugar content, cell damage and abnormal cell metabolism in wheat appear in seedlings treated with PAEs within 14 days [25,35]. Other effects include decreasing leaf biomass and shoot elongation and the accumulation of proline [4]. However, the toxicity of pollutants may be related not only to their contents in environmental media but also to their form and distribution in soils, soil nutrient content, mineral content, organic matter content, clay content and pH value. Diagnosis of soil contamination relying solely on chemical methods cannot comprehensively represent the overall quality and characteristics of the soil. Eco-toxicological diagnosis using toxicity tests on higher plants is an important method for diagnosing soil contamination ecologically and can provide qualitative or quantitative evaluation of adverse biological reactions to pollutants and provide important information on environmental protection [39].

The present study was conducted to investigate some key soil factors, namely soil pH, organic matter content and water content that influence the phytotoxic effects of two typical PAEs to a typical leafy vegetable. DnBP or DEHP was added singly at the beginning of lettuce transplanting for sub-chronic toxicity testing to compare their differences in toxicity during a cultivation period of 30 days by determining total protein, free amino acids and total soluble sugars, plant pigments (chlorophyll a and carotenoids), free radicals and ascorbic acid contents in plants, and effects on the soil microbiome. The results may be taken as recommendations regarding PAE toxicity and the utility of plastic films in greenhouse production under different soil conditions.

## Materials and methods

### Standards, assay kits and reagents

Di-n-butyl phthalate (DnBP, 99.1%) and di-(2-ethylhexyl) phthalate (DEHP, 99.6%) were obtained from AccuStandard, Inc., New Haven, CT. Certified reference material (CRM) 119–100 (BNAs—Sandy Loam 6) and 136–100 (BNAs—Clay 1) were purchased from RT Corporation (Laramie, WY, one of the original Proficiency Test providers recognized by USEPA). Assay kits (Catalog numbers A045-3 for BCA/total protein content, A009 for vitamin C and A052 for inhibition and production of superoxide radical) were purchased from Jiancheng Bioengineering Institute, Nanjing, east China. Sodium chloride, monopotassium phosphate, disodium hydrogen phosphate, sodium hydroxide, potassium chloride, potassium hydroxide, elhylene diamine tetraacetic acid, acetic acid, absolute ethyl alcohol and acetone were analytical grade reagents purchased from the National Pharmaceutical Group Chemical Reagent Co., Ltd., Shanghai, China.

### Test soil and plants

A typical ‘yellow brown’ soil classified as Alfisols according to the USDA soil classification system with a pH (in water) of 7.4 was collected from the top 15 cm of a relatively undisturbed mountain area (32° 8' 51" N, 118° 57' 58" E) at Qixia district, Nanjing, Jiangsu province, east China. No specific permissions were required for these locations/activities, because the sampling location is a barren mountain. The field studies did not involve endangered or protected species. The soil has a clay content of 1.67 g kg^-1^, an organic matter content of 1.46%, and available nitrogen, phosphorus and potassium concentrations of 96.8, 14.4 and 102.8 mg kg^-1^, respectively. The soil collected was passed through a 2-mm sieve prior to the incubation test and the soil background concentrations of DnBP and DEHP were determined to be 0.037 ± 0.002 and 0.087 ± 0.004 mg kg^-1^ (DW, oven dry basis).

Seeds of lettuce (*Lactuca sativa*) were obtained from the Chinese Academy of Agricultural Sciences in Nanjing. They were stored in a refrigerator at 4°C before use. Before being sown in prepared soil, seeds were surface sterilized by immersion in 10% sodium hypochlorite solution for 10 min, rinsing three times with deionized water, and soaking in deionized water for 2 h. Plastic equipment was avoided throughout the procedure to minimize PAE background contamination.

### Toxicity tests

Adjustment of the soil pH values to 7.0 and 8.5 was carried out ten days before sowing of the seeds by adding calcium hydroxide and sulfur powder. In addition, the organic matter contents in the different soil treatments were adjusted by adding peat at rates of 1.5, 3.0 and 4.5%. Soil water moisture was adjusted after the target pollutants were added. Ten treatments were set up as follows. (1) pH 7.0, 35% maximum water holding capacity (WHP) and 1.5% organic matter content (SOM); 2) pH 7.0, 60% WHP and 1.5% SOM; 3) pH 7.0, 85% WHP and 1.5% SOM; 4) pH 7.0, 60% WHP and 3.0% SOM; 5) pH 7.0, 60% WHP and 4.5% SOM; 6) pH 8.5, 35% WHP and 1.5% SOM; 7) pH 8.5, 60% WHP and 1.5% SOM; 8) pH 8.5, 85% WHP and 1.5% SOM; 9) pH 8.5, 60% WHP and 3.0% SOM; and 10) pH 8.5, 60% WHP and 4.5% SOM. Seven levels of soil contamination were prepared, namely the control (0 mg kg^-1^ PAE added) and 1, 5, and 20 mg DnBP kg^-1^ DW soil, and 1, 5 and 20 mg DEHP kg^-1^ DW soil and the soil was transferred to clay pots (2.0 kg per pot with four replicates). Soil PAE amendment was made by spraying with stock solutions in acetone (10 mL) before the seeds were sown and cultured for 30 days from March 20 to April 19 2017 at 25 ± 0.2°C under a light regime of 12-h days (4500 lux) with an air humidity of 80%. The same volume of acetone without PAE pollutants was used to prepare the 0 mg kg^-1^ DW control pots to account for solvent interference. After evaporation of the acetone each soil was mixed thoroughly with deionized water and the soil moisture was adjusted to 35, 60 or 85% of its maximum water holding capacity before use. Five lettuce seeds were sown in each clay pot after the soils were spiked. Soil moisture in all pots was maintained by weighing every day and mixing of the surface soil after watering to avoid different moisture contents at different depths in the pots. The seedlings were harvested and the soil was collected from each pot after 30 days.

### Quantitative analysis of PAEs

The analysis of DnBP or DEHP concentrations in soil after incubation for 30 days was carried out following the approach of Ma et al [40]. Target PAEs in 10 g of soil were extracted with a total volume of 70 mL acetone:hexane (1:1, v/v) three times in a water bath at 25°C and reduced in the flask by rotary evaporation to 1 ± 2 mL (350 mbar, 40°C water bath, 80 rpm) after centrifugation at about 252 ×*g*. Column chromatography purification was conducted in a glass column (1 × 26 cm) with 2g of Na_2_SO_4_, 6 g of neutral Al_2_O_3_ and 12 g of neutral silica gel (from bottom to top) with 55 ml of acetone:hexane (1:4, v/v) before collection and reduction to < 1 mL by rotary evaporation as described above.

Analysis was conducted with a 7890GC-5975 MSD gas chromatograph-mass spectrometer (Agilent, Santa Clara, CA). During analysis, whole procedure blanks, soil matrix blanks, spiked soil matrix and parallel samples were all employed together with analysis of a CRM sample to ensure quality control. The recovery rates of DnBP and DEHP extraction ranged from 80 to 110% and the recovery of the analysis method was 90.5% ± 4.1% which meets the requirements of trace PAE analysis in environmental materials.

### Determination of plant leaf area and biochemical indices

Measurement of leaf area was carried out using a handheld 3D laser scanner (HSCAN02, Scantech Co., Hangzhou, China) with the aid of the Geomagic image processing software package. After cutting into pieces and mixing thoroughly in an ice-cold bath, fresh plant samples of about 1.0 g were homogenized by grinding in ice-cold 50 mmol L^-1^ potassium phosphate buffer (PBS, pH 7.8) (w/v, 1:9) and centrifuged at 7104 ×*g* at 4°C for 20 min. The supernatant was used for further analysis of total protein content, •O_2_^-^ activity and vitamin C content using the corresponding assay kits. Total soluble sugar (TSS) content was estimated according to Moya et al [41] with slight modification. Eighty milliliters of 80% ethanol were added to 1.0 g of fresh sample and extracted in a water bath at 80°C for 30 min. The extraction was repeated twice and the mixture was centrifuged at 12 000 ×*g* for 10 min. All the supernatants were combined and made up to a final volume of 25 mL with 80% ethanol. Ten milliliters of extract were steamed to dryness over a boiling water bath, 0.5 mL of H_2_SO_4_-anthrone reagent was added and the TSS content was determined by spectrophotometry at 620 nm. Glucose was used as the standard for quantification. Free amino acid (FAA) content was estimated following the method of Shukla et al [42] with minor modifications to expand the test weight and volume in proportion. Fifty milligrams of ice-water bath ground fresh samples were stirred in 5 mL 10% acetic acid (v/v) solution overnight and distilled water was added to adjust the total volume to 50 mL. After filtration, 2 mL of the extract, 3 mL 1% ninhydrin (in 0.5 M citrate buffer, pH 5.5) and 0.1 mL 1% ascorbic acid were added and mixed before being heated in a boiling water bath for 15 min. After cooling, FAA content was determined by the absorbance at 570 nm. Alanine was used as the FAA standard. Samples (0.20 g) were homogenized in the dark in 95% ethanol (v/v) with a trace of calcium carbonate. The homogenate was centrifuged at 4°C, 7104 ×*g* for 20 min and the pigment content was calculated after the supernatant was measured spectrophotometrically at 665, 649 and 470 nm, respectively. The amounts of pigment were calculated using the equations of Ma et al [7].

### Determination of soil microbial community

For a simplified comparison of soil microbial community 12 groups of soil out of the designated 40 groups entitled S1 to S12 (Table 1) were screened for a Biolog test. The Biolog Eco test and the calculation of average well color development (AWCD) values, Shannon diversity index and McIntosh index were carried out following the methods of Ma et al [43] with the 12 soil samples listed in Table 1 using Gen5 v1.06 software.

**Table 1:** Soil sample treatments for the biolog test.

Sample number	PAE concentration (DW)	Other controlled variables
S1	0	Organic matter content 3.0%, Water content 60%, pH 7.0
S2	20 mg kg^-1^ DnBP	
S3	20 mg kg^-1^ DEHP	
S4	0	Organic matter content 3.0%, Water content 60%, pH 8.5
S5	20 mg kg^-1^ DnBP	
S6	20 mg kg^-1^ DEHP	
S7	0	Organic matter content 1.5%, Water content 35%, pH 7.0
S8	20 mg kg^-1^ DnBP	
S9	20 mg kg^-1^ DEHP	
S10	0	Organic matter content 1.5%, Water content 35%, pH 8.5
S11	20 mg kg^-1^ DnBP	
S12	20 mg kg^-1^ DEHP	

The Simpson index, weighted toward the abundances of the most common species, was used to emphasize the dominant population of soil microorganisms [44]. Simpson index was calculated as follows.

$$D=1-\sum_{i=1}^{n}p_{i}^{2}$$

where Pi is each reaction well subtracting the absorbance value of the control well and then dividing by the summed color absorbance value of 31 wells.

### Statistical analysis

All data were processed with Microsoft Excel 2013 and the SPSS v.18.0 software package. The data were analyzed for significant differences from the control or between treatments using one-way analysis of variance.

## Results

### Effects of target PAEs on leaf area and plant biomass

**Fig 1:**
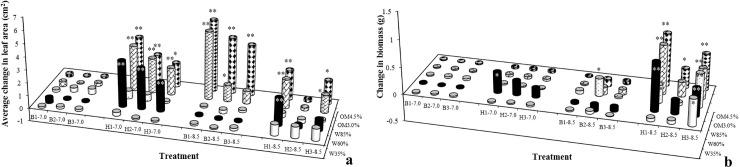
Effects of the target PAEs on (a) changes in lettuce leaf area and (b) changes in average biomass of the three largest leaves under different soil conditions. Each value is the mean of three replicates ± standard error of the mean (SEM). B, DnBP treatments; H, DEHP treatments; OM, soil organic matter content; W, % of maximum water holding capacity; B/H 7.0 or 8.5, the pH value of the test soil after addition of DnBP or DEHP, respectively. **, significant difference at *p* < 0.01; and *, significant difference at *p* < 0.05 compared with corresponding controls.

Effects of the target PAEs on leaf size under different soil physical and chemical conditions during the whole seedling growth period are shown in Fig 1A. The detailed leaf area values of the control treatments are shown in S1 Table. All leaf areas expanded under treatments B3-7.0, H1-7.0, B1-8.5, H1-8.5 and H3-8.5 (annotations as in Fig 1) compared with the controls. The most significant (*p* < 0.01) degree of increase was 5.58 cm^2^ in B1-8.5-OM4.5%, about one third of the area of its control. The leaf areas of all H-7.0-W60% treatments were significantly larger than those of the controls (*p* < 0.01). The results indicate that, under neutral soil conditions, an increase in lettuce leaf size occurred with increasing DEHP concentration compared with the controls. There was no discernible correlation between increasing leaf size and soil organic matter content across the different treatments with the two target pollutants. However, DnBP treatment was associated with a decline in lettuce leaf size compared with the control in about half of its treatments but not related with DnBP concentrations, soil water content or soil organic matter content, especially under neutral soil conditions. Under neutral soil conditions the soil water content is an important factor in the phytotoxicity of the two target PAE pollutants. Under alkaline soil conditions there were larger leaf sizes in both DEHP and DnBP treatments compared with the control. Increasing soil organic matter content significantly (*p* < 0.01) promoted leaf area at the different soil water contents but the effect was more pronounced in the DnBP treatments. Under alkaline soil conditions the organic matter content was a factor likely to affect the phytotoxicity of the two target PAE pollutants.

The effects of the target PAEs on plant biomass under different soil physical and chemical properties are shown in Fig 1B. In most treatments, plant biomass was promoted by the two target PAE compounds, especially under alkaline conditions in the presence of lower concentrations of DEHP at 60% water content, up to 0.968 g (*p* < 0.01). Under neutral conditions in DEHP treatments the increase in biomass was significant in only a few treatments (*p* < 0.05). However, in the DnBP treatments under different soil conditions almost all the plant biomass values showed no significant difference from the corresponding controls. In alkaline soil conditions soil water content was a factor likely to affect the phytotoxicity of DEHP. Biomass is not a sensitive indicator of DnBP toxicity.

### Phytochromes

**Fig 2:**
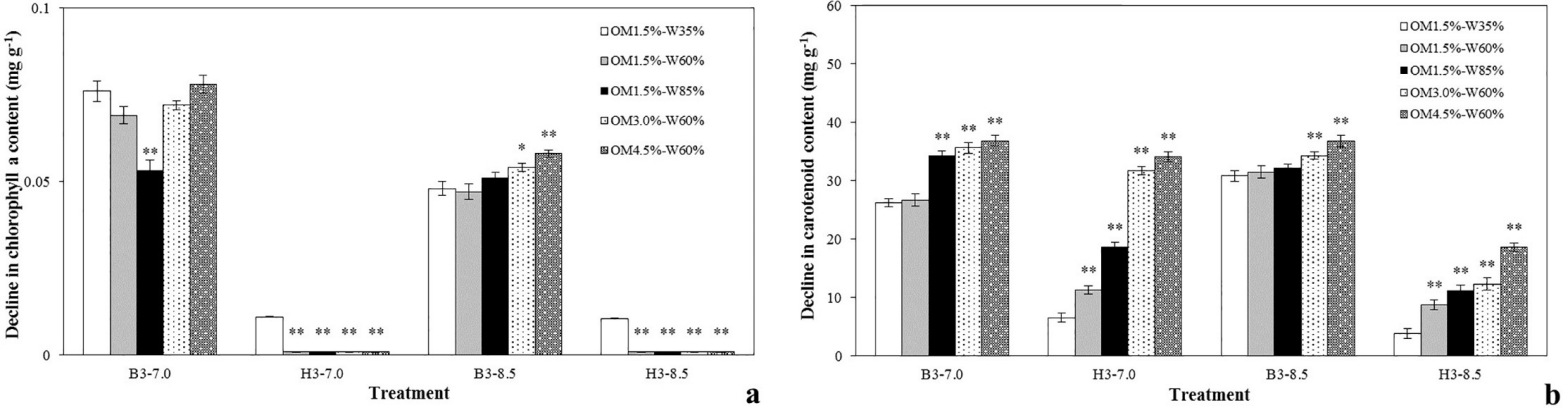
Effects of changes in (a) chlorophyll a and (b) carotenoid content at 20 mg kg^-1^ soil DW of two target PAEs under different soil conditions. Other annotations as in Fig 1.

Chlorophyll and carotenoids are important metabolites and play important roles in photosynthesis and the contents of plant pigments in the presence of the highest concentrations of the two target PAEs were determined after cultivation for 30 days. The results in Fig 2A and 2B show that similar declines occurred in the contents of chlorophyll a and carotenoids in lettuce leaves in the presence of the two target pollutants. The declines in chlorophyll a content (Fig 2A) in the DnBP treatments were almost five times higher than in the DEHP treatments in both neutral and alkaline soil conditions, and the differences compared with the control were all highly significant (*p* < 0.01). The decline in carotenoid content (Fig 2B) was clearly affected by the soilorganic matter content because significant differences were observed in treatments with 3.0% and 4.5% OM (*p* < 0.01), even at different soil pH levels. Carotenoids were greatly inhibited up to 36.8 mg g^-1^ by DnBP but leaf chlorophyll a content changed little in the presence of DEHP.

### Plant total protein, TSS and FAA contents

**Fig 3:**
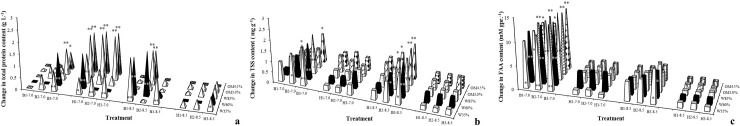
Effects of the target PAEs on changes in (a) foliar total protein content, (b) TSS and (c) FAA contents in lettuce under different soil conditions. Other annotations as in Fig 1.

The shoots are the most commonly consumed plant parts and the shoot total protein, TSS and FAA contents were determined here. All the contents of total protein showed different degrees of increase, especially in neutral DEHP and alkaline DnBP treatments without any correlation with soil water or organic matter contents (Fig 3A). The highest value was 1.898 g L^-1^, about 240 times of the lowest value (0.008 g L^-1^). The lower values occurred in alkaline soil treated with DEHP in which almost no significant differences were observed compared with the corresponding controls. Total protein contents were more likely to change with soil pH in the presence of either target pollutant.

TSS contents of lettuce shoots are summarized in Fig 3B. In a similar fashion to the total protein content results, the TSS contents of lettuce plants were all elevated. Significant promotion of TSS contents was observed in the presence of DnBP at different soil pH levels and this was accelerated by increasing pollutant concentrations in alkaline soils (*p* < 0.05). The highest value was 1.903 mg g^-1^, about six times the lowest value (0.312 mg g^-1^) in the DnBP treatments, while the highest value in the DEHP treatments was 0.785 mg g^-1^. However, DEHP had a much smaller effect on TSS contents under different soil conditions, and there were no significant differences among various water or organic matter contents between pollutant treatments and the control (*p* < 0.05). TSS contents in lettuce were affected more by soil pH than by the other soil properties examined.

Shoot FAA contents under different soil conditions are shown in Fig 3C. The FAA contents of the lettuce shoots also increased. At both soil pH values tested the FAA contents in neutral soil conditions increased with increasing DnBP addition but the effect was much smaller in alkaline soil. All the values in neutral soil treated with DnBP were significantly elevated (*p* < 0.05). However, in the presence of DEHP there were no significant differences from the corresponding controls (*p* < 0.05). Soil water content and OM content do not appear to have affected the results. Soil pH is therefore an important factor affecting the shoot FAA contents in the presence of the two target pollutants.

### Shoot •O_2_^-^ activity and vitamin C content

**Fig 4:**
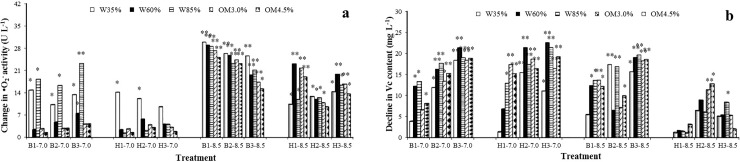
Effects of the target PAEs on changes in (a) activity of ·O_2_^-^ and (b) vitamin C contents of lettuce under different soil conditions. Other annotations as in Fig 1.

Increases in the superoxide anion radical contents in the shoots are shown in Fig 4A. The •O_2_^-^ activity was generally promoted in all treatments, in a similar fashion to the total protein, TSS and FAA contents. In the treatments under alkaline conditions the activities of •O_2_^-^ in leaves all increased significantly compared with the corresponding controls (*p* < 0.05). In neutral soil conditions, especially in the presence of DEHP, most of the activity values were not significantly elevated. However, the highest value (29.91 U L^-1^) was almost 30 times the lowest (1.21 U L^-1^).

Shoot vitamin C contents are shown in Fig 4B and were depressed in all treatments compared with the control. However, in the presence of both target pollutants there was a clearer increase in vitamin C content in neutral soil, because in almost every neutral soil treatment the vitamin C contents were significantly elevated (*p* < 0.05) but with no clear relationship with soil water or organic matter content. The highest value (22.56 mg L^-1^) was almost 22 times the lowest (1.03 mg L^-1^). Soil pH is therefore also more likely to affect the toxicity of the target PAEs.

### Soil microbial community

**Fig 5:**
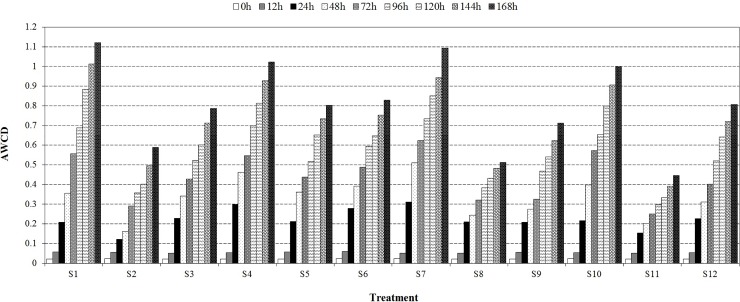
Effects of the target PAEs on AWCD values of treatments S1 to S12; results of substrate utilization potential of the soil microbial community under different soil conditions. Other annotations as in Table 1.

As shown in Fig 5, the AWCD values of all soil treatments increased with increasing time irrespective of soil pH or organic matter or water contents. Comparing the different treatments, AWCD values at 168 h generally followed the sequence S1 > S7 > S4 > S10 > S6 > S12 > S5 > S3 > S9 > S8 > S2 > S11. The AWCD values in the absence of the target PAEs were higher after incubation for 168 h with the sole exception of S12.

The effects of the target PAEs on the different soil microbial diversity indices are shown in Table 2. The Shannon, McIntosh and Simpson indices decreased when the target PAEs were added and the addition of DnBP gave higher inhibition of microbial diversity. The McIntosh index showed more pronounced differences than the other two indices.

**Table 2:** Effects of the target PAEs on soil microbial diversity *.

Sample number	Shannon Index	McIntosh index	Simpson index
S1	2.08±0.02a	4.38±0.13a	0.88±0.01a
S2	1.86±0.03a	3.15±0.15b	0.75±0.01a
S3	1.92±0.02a	3.51±0.12b	0.80±0.01a
S4	2.03±0.03a	4.12±0.08a	0.86±0.01a
S5	1.72±0.03b	3.23±0.12b	0.72±0.01a
S6	1.88±0.02a	3.63±0.11b	0.81±0.01a
S7	2.05±0.02a	4.21±0.18a	0.87±0.01a
S8	1.84±0.03b	3.18±0.09b	0.74±0.01a
S9	1.92±0.03a	3.52±0.13b	0.81±0.01a
S10	1.99±0.03a	4.17±0.12a	0.83±0.01a
S11	1.79±0.04b	3.13±0.14b	0.72±0.01a
S12	1.91±0.03a	3.39±0.12b	0.79±0.01a

*: Annotations as in Table 1. Different letters denote significant difference at *p* < 0.05 compared S2, S3 with S1; S5, S6 with S4; S8, S9 with S7; S11, S12 with S10.

## Discussion

Roots are generally more sensitive than shoots to soil pollutants because the roots are the main organ directly in contact with the soil and take up water and nutrients from the soil. However, the shoots are the edible part of leafy vegetables such as lettuce and physiological changes in the shoots may reflect plant growth though adverse impacts on photosynthesis and nutritional effects. We have therefore focused on the shoots in the present study.

### Plant biomass

Some of the environmental factors that may influence the degradation of toxic chemicals in the environment include soil pH, salinity, temperature, oxygen availability and nutrients [45]. Soil water content was the most important factor associated with the observed changes in leaf area in neutral soil conditions in the presence of DnBP or DEHP but soil organic matter content was more important in alkaline conditions. Plant biomass was a more suitable indicator of the toxicity of DEHP through significant effects (*p* < 0.05) and soil pH and water content were also important factors.

The degradation and adsorption of the target PAE pollutants were very different under neutral and alkaline soil conditions. Compounds with higher molecular weights and structural complexity, such as DEHP in this experiment, tend to be more resistant to biotransformation than those with lower molecular weights. Furthermore, because of differences in lipotropy the soil organic matter content may also affect the bioavailability of the target PAEs. PAEs are organic compounds with relatively low water solubility and are readily adsorbed onto soil organic matter rather than dissolved in the soil pore water when they are exerting their toxic effects [46]. Stimulatory effects on plant biomass by DEHP and inhibition by DnBP of different initial growth parameters have been observed in other plant species [47]. Stimulatory effects of low contents of environmental hormones on the growth and reproduction of different soil microorganisms have also been reported. Higher adsorption and lower bioavailability under alkaline soil conditions may have resulted in an increase in leaf area with even greater stimulation of leaf area under higher organic contents in the DEHP treatments. The expression of DEHP toxicity effects is vulnerable to external soil conditions, indicating higher toxicity of DnBP.

### Phytochromes

In both neutral and alkaline soil conditions the general trends of the test for both types of phytochrome in lettuce leaves show similar increases in the DnBP treatments and especially in the case of chlorophyll a, but little change occurred in the DEHP treatments.

Chlorophylls a and b are required for plant photosynthesis so that a decrease in chlorophyll contents will inevitably lead to a decline in photosynthetic capacity and hence the ability to synthesis organic matter and accumulate dry matter, all of which may lead to a decrease in plant biomass. In addition, carotenoids are natural pigments with very important functions in plants and in human health. Carotenoids are strong antioxidants that may delay the aging process of healthy cells, thus affecting the immune system, protecting the skin from damaging ultraviolet light and maintaining the information transmission stability and physiological functions of the cell [48]. Carotenoids can directly capture free radicals and block chain reactions of free radicals in order to prevent damage to proteins and lipids and peroxidation damage to DNA [49]. It has been reported that chlorophyll a + b and carotinoid contents were depressed in onion at all concentrations of PAEs examined [50]. Carotenoids may protect cells from mutagens and this is one of the mechanisms by which they can protect against cancers in consumers of vegetables. A decline in lettuce leaf carotenoid content therefore suggests a decrease in the ability of the plant to defend against free radicals in addition to a decrease in nutritional value and quality.

### Total protein, FAA and TSS contents

The plant total protein, FAA and TSS contents were the most sensitive factors reflecting the toxicity of the target PAEs because they changed significantly (*p* < 0.05) at different soil pH values. The free amino acid contents in the shoots in the presence of the pollutants increased more in neutral soil but the increases due to DnBP were more striking under alkaline soil conditions than those due to DEHP. Under different environmental pressures the resistance of the plant is expressed mainly by means of protein consumption and declining protein synthesis [7]. The changes in lettuce total protein contents may have been related to the inhibitory effects on biomass under the toxic influence of both PAE congeners. Overall, the toxicity effects differed under different soil pH conditions. The inhibition of total protein contents in the presence of DnBP under neutral soil conditions was clearly more pronounced than with DEHP under alkaline conditions under which plant biomass increased. Increasing biomass will inevitably lead to a decrease in the total protein contents and therefore DnBP toxicity to lettuce was more severe than that of DEHP because decreases in both biomass and total protein contents occurred. In general, when plants are subject to adverse environmental conditions such as drought, high salinity or low temperatures, TSS contents in the tissues will be promoted [51–53]. The reaction of the TSS content in the test lettuce treated with both PAEs indicates a slightly higher toxicity of DnBP than of DEHP, especially judging from the differences observed under alkaline conditions. FAA in muskmelon seedlings has been found to be induced even by low doses of trifluralin, similar to the increase in TSS contents found under unfavorable environmental conditions [51–53]. During the seedling growth period the promotion of total protein, TSS and FAA contents is a distinct sign of environmental pressure due to the two target PAEs in the current study.

### Leaf •O_2_^-^ activity and plant vitamin C content

The activity of •O_2_^-^ in the lettuce leaves was sensitive to the pH value of the soil when treated with both target PAE pollutants. Aerobic metabolism is necessary for the survival of higher plants. Energy for plant growth and development can be provided by the reduction of oxygen to form water. However, incomplete reduction will produce reactive oxygen species (ROS) which have strong oxidation capacity and include •O_2_^-^, the hydroxy radical, singlet oxygen and hydrogen peroxide.

•O_2_^-^ is one of the most important free radicals produced during the oxidation process in plants under the stress of toxic substances and may lead to a loss of membrane structure and damage to important plant pigments of photosynthesis and the photosynthetic system [54]. Moreover, •O_2_^-^ can be transformed into more harmful substances such as the hydroxyl radical and hydrogen peroxide which may accumulate and impair the photosynthetic system and many biologically active molecules in plants by triggering or exacerbating membrane lipid peroxidation, and thus lead to considerable damage to the structure of, or degrade the function of, the cell membrane system, or cause metabolic disturbance [55]. The detection of •O_2_^-^ in plants can increase our understanding about reactions of the plant against environmental stress. Under normal conditions the oxygen scavenging system can effectively scavenge active oxygen free radicals in plants to protect the cells from damage. However, faced with adverse environmental conditions it will cause damage when the production rate of plant active oxygen free radicals is beyond the ability of the plant to eliminate active oxygen [56,57]. In this toxicity test the significant increase (*p* < 0.05) in •O_2_^-^ activity in lettuce under DnBP treatment indicates severe toxicity damage due to the pollutant via the production of oxygen free radicals, especially in alkaline soil conditions.

Plant vitamin C content was significantly (*p* < 0.05) inhibited compared with the control in most treatments but was slightly promoted in some DEHP treatments. Vitamin C, (ascorbic acid) is a small molecule that is very important in higher plant cells for the prevention of oxidative stress, in cell division and in cell elongation [58]. It is synthesized in the mitochondria and then transported to other cellular compartments. Vitamin C plays an important role in disease resistance as a molecular resistance signal in addition to its regular biological functions and participation in adaptation and resistance to adverse conditions. It also participates in the regulation of plant cell oxidation reduction equilibrium as an important redox buffer, regulates the transcription and translation of some genes, regulates the activities of some enzymes as a coenzyme, participates in the synthesis, elongation and crosslinking of plant cell walls, and regulates the division and elongation of the cells. The decline in the vitamin C content of lettuce in our test indicates that the test plants were under environmental stress and the vitamin was consumed for various defensive processes. On the other hand, the decline in lettuce vitamin C content under the toxicity of the two target PAEs also involves a decline in the nutritional value of the vegetable, a similar outcome to the decline in carotenoid pigments. Soil pH is also the most important factor affecting the toxicity of the target PAEs.

### Soil microbial community

The Biolog Eco plate analysis indicates that microbial activities were generally inhibited by addition of the target PAEs at a concentration of 20 mg kg^-1^ DW and the degree of inhibition was more pronounced in the DnBP treatments (Fig 5 and Table 2). Microbial diversity indices were also influenced by the target PAEs. Low contents of PAEs have a stimulatory effect on the growth of different soil microorganisms [43] but disruption occurs at higher concentrations, greatly affected by soil pH, followed by soil organic matter content and finally the soil water content. In PAE-contaminated soil at higher pH values there may have been less harmful and less toxic effects, indicating higher vegetable safety in alkaline soil conditions.

### Influence of soil physicochemical properties on the test indices of lettuce and soil microbial community

Soil pH is likely to be the most important factor in the toxicity of DnBP and DEHP to lettuce. Under neutral soil conditions the leaf area, the total protein content in DEHP treatments, the FAA content in DnBP treatments, the activity of •O_2_^-^ in leaves in DEHP treatments and the vitamin C content in treatments with both pollutants changed greatly and often significantly (*p* < 0.05 or 0.01). However, under alkaline soil conditions the leaf area was also impacted by soil organic matter content. In contrast to the biomass results in DEHP treatments, soil water content was a factor likely to affect the phytotoxicity of DEHP, although biomass is not a sensitive indicator of DnBP toxicity. In alkaline soil conditions leaf total protein and TSS contents were more affected by DnBP, but with no clear correlation with soil water content or organic matter content. DEHP had little effect on TSS contents under different soil conditions, so that pH values had little influence, a similar trend to that of FAA in DEHP treatments. carotenoid content clearly declined in response to increasing soil organic matter content at both soil pH levels investigated. In conclusion, most of the plant toxicity indicators tested were influenced more by soil pH than by soil organic matter or water content.

### PAE dosage and effects on lettuce

The original soil collected for spiking had an organic matter of 1.46% and a pH of 7.4 and this is most similar to treatment 2. The dose-effect relationships between the target pollutants and the plants were therefore examined (S2 Table) and the plant and soil concentrations of DnBP and DEHP at harvest are listed in S3 Table. Across the seven toxicity indicators determined, only TSS content treated showed a highly correlated change in response to both PAEs, indicating a significant increase in TSS with increasing soil DnBP or DEHP concentration. Negative linear correlations were found in changes in leaf area and biomass in DEHP treatments and positive linear correlations in changing total protein content, FAA content, •O_2_^-^ activity and vitamin C content in DnBP treatments. Regression analysis in another study shows highly significant inhibition of mung bean root elongation (*p* < 0.01) under exposure to both DnBP and DEHP, highly significant retardation of shoot elongation (*p* < 0.01) under DEHP and significant depression of biomass (*p* < 0.05) by DnBP during the germination period [59]. During the germination of rape seedlings DnBP showed a negative significant correlation (*p* < 0.01) with root elongation, shoot elongation and biomass, while DEHP showed a negative significant correlation (*p* < 0.05) with shoot elongation but positive correlations with root elongation and biomass [50]. The vitamin C content in cucumber was negatively affected (*p* < 0.05) by DnBP treatment but the soluble sugar content (*p* < 0.01) showed the opposite trend. The range of results in different plant species indicates a range of mechanisms in different plants when confronted with environmental stress.

Many agricultural soils and vegetables in China are contaminated with PAEs [13–21] but the control and remediation of PAE contamination have not attracted much public concern. Investigations such as that conducted in the present studies may serve as an alert to this degradation of soil quality including potential impacts on soil microorganisms and on food quality. More detailed soil PAE environmental quality standards are required to enforce soil remediation and minimize soil toxicity. The establishment of detailed quality inspection standards for agricultural products in respect of PAE pollutants is also strongly recommended [60, 61].

## Conclusions

Soil pH is likely to be an important factor affecting leaf area, biomass, total protein content, TSS, FAA content, other foliar biochemical indicators and the soil microbial community in the presence of contamination with DnBP or DEHP. However, under neutral soil conditions soil water content is also an important factor involved in the phytotoxicity of the two target PAE pollutants. Under alkaline conditions soil organic matter content is important and soil water content affects the phytotoxicity of DEHP. Comparing all seven plant indicators and the kinetic equations of the two PAE pollutants, DnBP was found to be more toxic than DEHP, with a ratio of 5:3 in significant effect (*p* < 0.05) in dose-effect analysis of the soil. Many of the soils in north China are calcareous but acid soils predominate in south China and this may lead to differences in the importance of different PAE pollutants in agricultural soils in different regions. Further studies are required to understand and minimize soil PAE pollution and to ensure food safety on a national scale.

## Supporting information

S1 Table (DOCX)

S2 Table (DOCX)

S3 Table (DOCX)

S1 File All data. (XLSX)
