# Supplementary material for: Toxicity of phthalate esters to lettuce (Lactuca sativa) and the soil microbial community under different soil conditions
Source: PLoS One. 2018 Dec 20;13(12):e0208111. doi: 10.1371/journal.pone.0208111 (PMC6301558; doi:10.1371/journal.pone.0208111)
Supplement: S1 Table — (DOCX) [file pone.0208111.s001.docx]

**Table S1 Determined Values of All Twelve Controls for All Test Parameters.**

| Treatment | Leaf area / cm^2^ | Biomass / g | chlorophyll a content / mg g^-1^ | carotenoid content / mg g^-1^ | Total protein content / mg g^-1^ | TSS content / mg g^-1^ | FAA content / mM gpr.^-1^ | ·O_2_^-^ activity / U L^-1^ | vitamin C contents/ mg L^-1^ |
| --- | --- | --- | --- | --- | --- | --- | --- | --- | --- |
| 1-control | 19.66±0.92 | 9.13±0.92 | 0.49±0.02 | 123.38±0.33 | 10.87±0.56 | 15.98±0.22 | 20.71±0.57 | 113.26±0.59 | 155.69±0.59 |
| 2-control | 20.17±1.33 | 9.33±0.52 | 0.52±0.03 | 127.52±0.37 | 11.34±0.37 | 16.33±0.29 | 21.28±0.33 | 133.52±0.98 | 158.85±0.76 |
| 3-control | 20.08±1.17 | 9.28±0.69 | 0.47±0.02 | 122.28±0.23 | 10.98±0.51 | 16.13±0.31 | 21.19±0.25 | 128.51±0.69 | 156.36±0.56 |
| 4-control | 21.08±1.13 | 9.27±0.33 | 0.48±0.02 | 121.77±0.28 | 10.72±0.39 | 15.88±0.37 | 20.39±0.24 | 131.73±0.67 | 154.59±0.83 |
| 5-control | 21.77±1.32 | 9.56±0.85 | 0.47±0.03 | 120.28±0.34 | 11.01±0.54 | 15.97±0.40 | 20.54±0.32 | 132.82±0.75 | 155.29±0.54 |
| 6-control | 17.11±1.41 | 9.68±1.08 | 0.49±0.03 | 118.93±0.54 | 11.21±0.47 | 15.85±0.36 | 21.15±0.41 | 130.25±0.51 | 152.47±0.48 |
| 7-control | 18.83±1.12 | 10.07±1.10 | 0.48±0.04 | 122.39±0.41 | 11.75±0.63 | 15.86±0.59 | 20.89±0.88 | 131.23±0.69 | 156.37±0.64 |
| 8-control | 18.99±1.42 | 9.93±1.15 | 0.44±0.03 | 117.75±0.56 | 10.99±0.37 | 15.95±0.51 | 20.58±0.34 | 131.58±0.36 | 156.87±0.73 |
| 9-control | 18.96±1.47 | 9.69±1.13 | 0.48±0.03 | 118.59±0.64 | 11.51±0.41 | 15.97±0.36 | 20.94±0.34 | 133.25±0.76 | 152.68±0.76 |
| 10-control | 19.56±1.26 | 10.02±0.99 | 0.46±0.02 | 117.56±0.42 | 11.26±0.48 | 16.02±0.23 | 21.17±0.36 | 130.59±0.63 | 156.26±0.82 |

Annotations as in “Toxicity test”. Each value is the mean of the largest three leaves ± standard error of the mean (SEM).
