# Supplementary material for: Toxicity of phthalate esters to lettuce (Lactuca sativa) and the soil microbial community under different soil conditions
Source: PLoS One. 2018 Dec 20;13(12):e0208111. doi: 10.1371/journal.pone.0208111 (PMC6301558; doi:10.1371/journal.pone.0208111)
Supplement: S2 Table — (DOCX) [file pone.0208111.s002.docx]

**Table S2 Dosage and Effects Between Pollutant Concentrations and the Effects in Test Lettuce.**

| Treatment | DnBP treatment | | DEHP treatment | |
| --- | --- | --- | --- | --- |
|  | Kinetic equation | *R* | Kinetic equation | *R* |
| Change in leaf area | y = 0.0101x - 0.0245 | 0.6390 | y = -0.0667x + 3.5879 | ***0.9948*** |
| Change in biomass | y = 5E-05x + 0.0142 | 0.1655 | y = -0.0098x + 0.3569 | ***0.9246*** |
| Change in total protein content | y = 0.0775x - 0.2041 | ***0.9805*** | y = -0.0527x + 0.9944 | 0.6544 |
| Change in TSS content | y = 0.0644x + 0.4365 | ***0.9472*** | y = 0.0221x + 0.3114 | ***0.9990*** |
| Change in FAA content | y = 0.1104x + 11.2 | ***0.8936*** | y = 0.0143x + 3.7624 | 0.6458 |
| Change in •O_2_^-^ activity | y = 0.2447x + 2.7126 | ***0.9636*** | y = 0.0371x + 3.6555 | 0.2216 |
| Change in Vc content | y = 0.443x + 12.738 | ***0.9676*** | y = 0.6264x + 11.462 | 0.7115 |

*R* < 0.4, the linear correlation is low; 0.4≤ *R* <0.7, the linear correlation is medium; 0.7≤ *R* <1, the linear correlation is high.
