# Supplementary material for: Toxicity of phthalate esters to lettuce (Lactuca sativa) and the soil microbial community under different soil conditions
Source: PLoS One. 2018 Dec 20;13(12):e0208111. doi: 10.1371/journal.pone.0208111 (PMC6301558; doi:10.1371/journal.pone.0208111)
Supplement: S3 Table — (DOCX) [file pone.0208111.s003.docx]

**Table S3** **Concentrations of DnBP and DEHP in Lettuce and Soil at Harvest (mg kg^-1^)**

| Treatment | DnBP concentration in lettuce | | | | DEHP concentration in lettuce | | | | Soil DnBP concentration | | | | Conc. of DEHP in soil | | | |
| --- | --- | --- | --- | --- | --- | --- | --- | --- | --- | --- | --- | --- | --- | --- | --- | --- |
|  | 0 | 1 | 5 | 20 | 0 | 1 | 5 | 20 | 0 | 1 | 5 | 20 | 0 | 1 | 5 | 20 |
| 1 | - | 0.227±0.003 | 0.582±0.008 | 1.307±0.018 | - | 0.236±0.004 | 0.638±0.017 | 1.896±0.133 | - | 0.722±0.007 | 4.228±0.017 | 17.233±0.013 | - | 0.901±0.006 | 4.733±0.021 | 18.971±0.038 |
| 2 | - | 0.219±0.004 | 0.554±0.012 | 1.285±0.021 | - | 0.224±0.005 | 0.693±0.018 | 1.989±0.141 | - | 0.713±0.008 | 4.233±0.018 | 17.171±0.017 | - | 0.899±0.007 | 4.565±0.022 | 19.338±0.027 |
| 3 | - | 0.221±0.004 | 0.539±0.007 | 1.331±0.019 | - | 0.232±0.004 | 0.622±0.021 | 1.898±0.133 | - | 0.726±0.007 | 4.528±0.017 | 17.197±0.011 | - | 0.906±0.005 | 4.598±0.018 | 19.216±0.021 |
| 4 | - | 0.230±0.006 | 0.518±0.011 | 1.298±0.023 | - | 0.241±0.007 | 0.619±0.019 | 1.831±0.127 | - | 0.718±0.009 | 4.371±0.017 | 17.747±0.018 | - | 0.917±0.008 | 4.602±0.017 | 19.514±0.033 |
| 5 | - | 0.228±0.005 | 0.515±0.013 | 1.373±0.025 | - | 0.229±0.005 | 0.623±0.018 | 1.852±0.118 | - | 0.716±0.011 | 4.338±0.014 | 17.235±0.021 | - | 0.921±0.007 | 4.617±0.017 | 19.532±0.036 |
| 6 | - | 0.225±0.002 | 0.562±0.015 | 1.392±0.018 | - | 0.263±0.007 | 0.620±0.018 | 1.879±0.132 | - | 0.722±0.009 | 4.196±0.021 | 17.228±0.017 | - | 0.911±0.007 | 4.663±0.021 | 19.253±0.020 |
| 7 | - | 0.218±0.004 | 0.573±0.018 | 1.286±0.015 | - | 0.256±0.005 | 0.636±0.016 | 1.856±0.121 | - | 0.735±0.007 | 4.117±0.027 | 17.914±0.020 | - | 0.918±0.009 | 4.612±0.019 | 19.268±0.023 |
| 8 | - | 0.220±0.003 | 0.588±0.013 | 1.309±0.018 | - | 0.259±0.004 | 0.657±0.019 | 1.882±0.130 | - | 0.743±0.008 | 4.194±0.017 | 17.356±0.017 | - | 0.939±0.006 | 4.613±0.023 | 19.278±0.031 |
| 9 | - | 0.222±0.004 | 0.593±0.018 | 1.322±0.021 | - | 0.248±0.007 | 0.629±0.019 | 1.897±0.142 | - | 0.727±0.010 | 4.277±0.018 | 17.347±0.021 | - | 0.928±0.011 | 4.620±0.021 | 19.387±.027 |
| 10 | - | 0.224±0.003 | 0.586±0.012 | 1.338±0.020 | - | 0.236±0.005 | 0.631±0.017 | 1.785±0.126 | - | 0.715±0.007 | 4.291±0.021 | 17.312±0.021 | - | 0.914±0.010 | 4.745±0.017 | 19.232±0.019 |

-, concentration < 0.1 mg kg^-1^. Annotations as in “Toxicity test”. Each value is the mean of four replicate pots ± standard error of the mean (SEM).
